# Effects of metformin and exercise training, alone or in association, on cardio-pulmonary performance and quality of life in insulin resistance patients

**DOI:** 10.1186/1475-2840-13-93

**Published:** 2014-05-15

**Authors:** Christian Cadeddu, Silvio Nocco, Cugusi Lucia, Martino Deidda, Alessandro Bina, Orru Fabio, Stefano Bandinu, Efisio Cossu, Marco Giorgio Baroni, Giuseppe Mercuro

**Affiliations:** 1 Department of Medical Sciences “M. Aresu”, University Hospital of Cagliari, SS 554, Km 4.500, 09042 Monserrato, Italy

**Keywords:** Insulin resistance, Cardiopulmonary exercise test, Metformin, Exercise training, Quality of life

## Abstract

**Background:**

Metformin (MET) therapy exerts positive effects improving glucose tolerance and preventing the evolution toward diabetes in insulin resistant patients. It has been shown that adding MET to exercise training does not improve insulin sensitivity. The aim of this study was to determine the effect of MET and exercise training alone or in combination on maximal aerobic capacity and, as a secondary end-point on quality of life indexes in individuals with insulin resistance.

**Methods:**

75 insulin resistant patients were enrolled and subsequently assigned to MET (M), MET with exercise training (MEx), and exercise training alone (Ex). 12-weeks of supervised exercise-training program was carried out in both Ex and MEx groups. Cardiopulmonary exercise test and SF-36 to evaluate Health-Related Quality of Life (HRQoL) was performed at basal and after 12-weeks of treatment.

**Results:**

Cardiopulmonary exercise test showed a significant increase of peak VO2 in Ex and MEx whereas M showed no improvement of peak VO2 (∆ VO2 [CI 95%] Ex +0.26 [0.47 to 0.05] l/min; ∆ VO2 MEx +0.19 [0.33 to 0.05] l/min; ∆ VO2 M -0.09 [-0.03 to -0.15] l/min; M vs E p < 0.01; M vs MEx p < 0.01; MEx vs Ex p = ns). SF-36 highlighted a significant increase in general QoL index in the MEx (58.3 ± 19 vs 77.3 ± 16; p < 0.01) and Ex (62.1 ± 17 vs 73.7 ± 12; p < 0.005) groups.

**Conclusions:**

We evidenced that cardiopulmonary negative effects showed by MET therapy may be counterbalanced with the combination of exercise training. Given that exercise training associated with MET produced similar effects to exercise training alone in terms of maximal aerobic capacity and HRQoL, programmed exercise training remains the first choice therapy in insulin resistant patients.

## Introduction

Insulin resistance (IR), a condition in which normal levels of this hormone produce a sub-optimal biological response, is considered a primary etiologic factor in the development of type 2 diabetes (T2DM), ischemic heart disease but also non-ischemic heart failure [1]. As a matter of fact IR is associated with a form of cardiomyopathy in which the myocardium is incapable of responding to injuries by altering substrate metabolism to increase energy efficiency. Moreover, recent studies have shown that IR plays an important role in determining a reduction in cardiopulmonary (CP) performance, intended as peak oxygen uptake, anaerobic threshold, workload and specific pulmonary function parameters as VE/VCO2 [2]. Lately, IR has been able to predict the subsequent development of heart failure, regardless of all the known risk factors, including T2DM [3,4].

The U.S. Diabetes Prevention Program (DPP) demonstrated that lifestyle modification (i.e., low-fat diet and increased physical activity) and the insulin sensitizer medication Metformin (MET) reduced the rate of transition from pre-diabetes to T2DM [5]. The American Diabetes Association strongly recommends exercise as a cornerstone therapy for diabetes prevention and, recently, suggested that some individuals with pre-diabetes be considered for MET treatment [6,7]. However, MET has also been shown to slightly but significantly reduce oxygen consumption in a population of healthy individuals [8]. Also, MET reduces average oxygen consumption to a modest but significant extent in IR subjects. Indeed, this effect is not manifested by all subjects, being present only in patients featuring a lower degree of IR, while in patients with very high IR, CP performance is significantly improved [9,10]. In an heart failure setting, Wong et al. showed how metformin did not increase peak VO2 but reduced the sub-maximal measure of VE/VCO2 slope in patients affected by heart failure and IR [11].

Malin et all showed how exercise training and MET are both able to improve insulin sensitivity after 12 weeks of therapy in men and women with pre-diabetes. But subtle differences among condition means suggest that by adding MET the full effect of exercise training is blunted [12].

Moreover previous studies have shown that T2DM and impaired glucose tolerance (IGT) was significantly related with low health-related quality of life (HRQoL) [13]. In addition, more recently studies on individuals with pre-diabetes showed that patients who were more physically active had better physical and mental HRQoL than patients who were inactive. Furthermore the lifestyle modification characterized by intentional weight loss and increased physical activity has an association with better HRQoL in overweight or obese participants at high risk for T2DM [14,15].

There is considerable need to better understand the potential for additive effects of physical activity when MET is used concurrently in terms of CP performance and HRQoL. The purpose of this study was to compare the effect, of exercise training and MET alone or in combination, on maximal aerobic capacity and, as a secondary end-point on quality of life indexes in IR patients.

## Methods

### Study population

Seventy five patients (35 males and 40 females; 46 ± 11 years), consecutively selected from a population of individuals screened at the Diabetic Centre at our University Hospital, were enrolled on the study from Jan 2009 till Dec 2010. All enrolled patients were white Caucasian and lived in the geographic area of south Sardinia. All patients presented impaired glucose tolerance (IGT) which had been recently identified, and/or impaired fasting glucose (IFG), and all were affected by IR, calculated in accordance with the homeostatic model assessment (HOMA) index and defined according to the values of Bonora et al. [16,17].

Inclusion criteria for both IR patients were: age 20–55 years, echocardiographic left ventricle ejection fraction (LVEF) ≥55% and absence of echocardiographic wall motion abnormalities, normal hepatic and renal function (bilirubin ≤1.5 mg/dl, creatinine ≤2.0 mg/dl). Exclusion criteria were diabetes, smoking, hypertension with left ventricle hypertrophy, moderate to severe heart valve disease, atrial fibrillation or severe arrhythmias. The presence of dyslipidemia or hypertension without left ventricle hypertrophy was admitted. The present study was approved by the Ethical Committee of our University Hospital of the AOU of Cagliari and informed written consent was obtained from all participants.

### Study protocol

**Figure 1:**
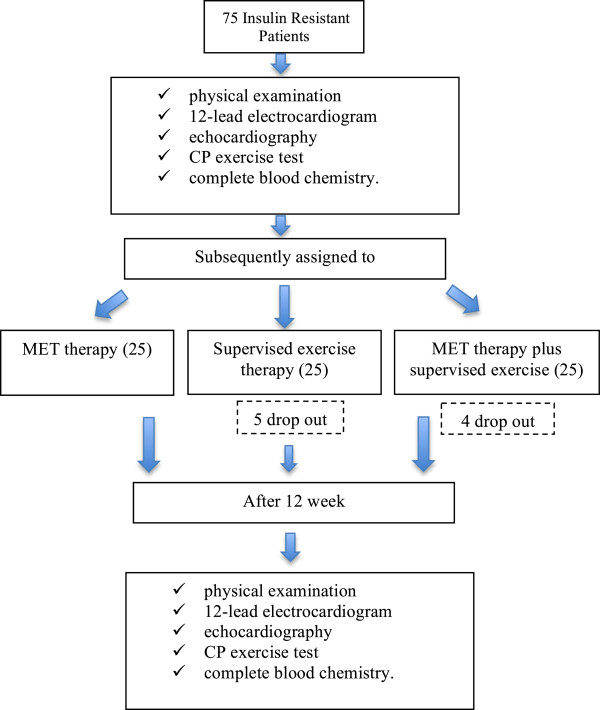
Flow chart on the study design.

The study was spontaneous, not sponsored and blinded to investigators. At enrolment, all subjects underwent physical examination, 12-lead electrocardiogram, M-mode, 2D and Doppler-echocardiography, CP exercise test (CPET) and complete blood chemistry. All patients were subsequently assigned to the 3 treatment groups: Group M (n 25) assigned to 12 weeks of MET therapy, Group Ex (n 25) assigned to 12 weeks of supervised exercise therapy, Group MEx (n 25) assigned to 12 weeks of MET therapy plus supervised exercise (Figure 1). The subjects of the groups MET and MEx started treatment with 500 mg/day of metformin. The dose was increased 500 mg/day after the first week to the clinical dose of 1,000 mg/day. Subjects remained at this dose for the last 11 weeks remaining. All the enrolled patients were re-evaluated after 12 weeks.

Primary endpoint of the study was prospectively defined as a significant increase of Vo2 max and secondary endpoint was the improvement of HRQoL.

### Conventional echocardiography

Echocardiographic images were recorded using a commercially available system (Toshiba Artida; Toshiba Corp., Tochigi, Japan). LVEF was obtained from the apical 4- and 2-chamber views according to Simpson’s rule and was considered abnormal when <55%. Pulsed wave Doppler (PWD) examination was performed of the left ventricle inflow from the 4-chamber view with the sample volume placed between the mitral leaflet tips and the early (E) and late (A) diastolic peak velocities; E deceleration time (DecT) was measured and the E/A ratio was derived.

### Cardiopulmonary exercise test

All subjects underwent an integrated maximal CP exercise test on an electrically braked stationary cycle ergometer. Patients wore a tightly fitting facemask connected to a capnograph and a sample tube enabling online ventilation and metabolic gas exchange computation. The calibrations of the tachometer and of oxygen sensors and carbon dioxide were carried out before each test. Heart rate and rhythm were continuously monitored using a 12-lead ECG, recorded every 30 seconds during exercise and a 10-minute post-exercise recovery period. Arterial blood pressure was ascertained using the standard cuff technique with a mercury sphygmomanometer placed on the left arm. Measurements were obtained every 3 minutes during exercise and at the 1st, 5th, and 10th minute of recovery.

At baseline, patients breathed slowly for a minimum of 5 minutes to stabilize resting gas measurements. A ramp protocol with an exercise regimen of a 3-min warm-up at 10 W at a pedal speed of 60–65 revolutions/min was applied, followed by a linear increase in the workload at a rate of 1 W every 6 s (10 W/min). In this way, at least 10 minutes exercise for each patient was obtained. Patients were strongly encouraged to continue as long as possible and reach muscular exhaustion. Breath-by-breath VO2, carbon dioxide production (V CO2), and minute ventilation (VE) were measured throughout the test using Vmax C software (Sensormedics, Yorba Linda, CA). The end point of the CPET test was determined as per the guidelines for diagnostic evaluation of patients with chronic ischemic heart disease. Peak VO2 and the consumption of oxygen at anaerobic threshold were expressed as an absolute value, standardized for weight and in percentage compared to the above-mentioned values according to the Wasserman formula [18]. Anaerobic threshold was calculated by two independent skilled operators using the V-Slope method and compared with values obtained by graphs VE/VO2 – VE/VCO2 (relationship between ventilation and oxygen consumption and between ventilation and production of carbon dioxide) and PetO2 - PetCO2 (concentration of O2 and CO2 at the end of exhalation). At the peak of exercise the pulse of oxygen (consumption between oxygen standardized for the heart rate) and breathing quotient (relationship between CO2 e O2) were calculated. Subsequently, curve slopes VO2/Work and VE/VCO2, the latter excluding the final non-linear portion of the curve, were elaborated.

### Health related quality of life assessment

HRQoL was assessed using the Medical Outcome Study 36-item short form health survey (SF-36) questionnaire [19]. The Italian-language version of the SF-36 is a validated instrument [20] containing 36 items divided into eight dimensions of health using multi-item scales: Physical functioning, role limitations due to physical functioning (Role-physical), Bodily pain, Mental health, role limitations due to emotional functioning (Role-emotional), Social functioning, Vitality, and General Health perceptions. The eight scales were scored from 0 to 100 (worst to best possible health status). For each dimension, the score represents the mean of item values obtained by the subject when all the items were completed or when the number of missing values was no more than half of the total items. As described elsewhere Physical (PCS) and Mental (MCS) Component Summary assessing the impact of health on physical and social/emotional function, respectively [19,20].

### Exercise training program

The same exercise-training program was carried out in both exercise groups (Ex and MEx). The training protocol consisted of a heating phase, a central and a cool-down phase with stretching exercises. The heating phase (10 minute) consisted of light stretching, walking and mobility of trunk, limbs and arms. The central phase consisted in 30 to 50 minutes of cycle ergometer training. Each session was conducted at a specific intensity target, with a heart rate range from 60% to 80% of the heart rate reserve (HRR) based on the subject’s age. Finally, a cool-down period, consisting of 10 minutes of mobility exercises, muscular relaxation and stretching was performed.

Six weeks after starting the training program, each subject underwent a graded exercise test to readjust the training workload [21].

### Statistical analysis

A sample size of 17 subjects per arm has been calculated sufficient, based on our previous studies on the effects of metformin on VO2 max using CPET [9]. For anthropometric and clinical characteristics of the two groups, continuous variables were compared with Analysis of Variance (ANOVA), and categorical variables were compared with Fisher’s exact test. Differences in echocardiographic parameters were also evaluated using ANOVA. A two-tailed value of p < 0.05 was considered statistically significant. For the multiple statistical test the Bonferroni correction had been applied. A “per protocol” analysis was performed.

## Results

The global population enrolled on the study showed an average HOMA of 5.48 ± 3.8 and among them mainly overweight or frankly obese (Table 1). CPET showed in all the enrolled patients a reduced average peak VO2 (61.8% ± 12%) compared with the theoretical values of a normal population paired for age and anthropometric characteristics (Table 1).

**Table 1:** Clinical features of study population

Age	46.2 ± 11	Glycaemia (mg/dl)	112 ± 14	***CPET parameters***	
Height (cm S)	167.2 ± 9	Insulinaemia(μU/m)	23.7 ± 14.1	VO2 peak (ml/kg/min)	20.16 ± 3.72
Weight (Kg S)	83.3 ± 11	HOMA	5.48 ± 3.8	%	61.83 ± 12
BMI (Kg/m^2^)	29.8 ± 4.1	***Glycemic profile***		VO2 (l/min)	1.70 ± 0.41
Total cholesterol (mg/dl)	206 ± 14	NG (%)	17%	%	73.6 ± 12.3
HDL (mg/dl)	52 ± 10	IFG (%)	71%	WORK (Watt)	114.6 ± 31
LDL (mg/dl)	131 ± 24	IGT (%)	28%	AT (l/min)	1.01 ± 0.25
Triglycerides (mg/dl)	146 ± 55	IGT + IFG (%)	22%	AT% VO2 peak	48.1 ± 11
Hypertension (%)	34.7%	Diabetes (%)	0%	VO2/WORK	9.7 ± 1.36

*Abbreviations*: *BMI* Body Mass Index, *HOMA* Homeostasis Model Assessment, *VO2peak* Maximum oxygen uptake, *% VO2peak* Maximum oxygen uptake in percentage compared to normal values, *Work* Maximum work, *AT* Anaerobic threshold,* % AT* Anaerobic threshold in percentage compared to VO2peak, *VO2/Work* Oxygen uptake-Work rate relationship.

No differences in term of insulin sensitivity, cardiovascular risk factors and medications taken have been evidenced between groups (Tables 2, 3).

**Table 2:** Risk factors and treatment in the 3 groups

	**Metformin**		**Exercise Training**		**Metformin and Exercise Training**	
Smoke	4	16%	5	20%	5	20%
Hypertension	9	36%	12	48%	8	32%
Hyperchol.	7	28%	8	32%	6	24%
CAD	0	0%	0	0%	0	0%
PAD	0	0%	0	0%	0	0%
Therapy						
BB	0	0%	0	0%	0	0%
ACEi	5	20%	7	28%	3	12%
ARB	3	12%	2	8%	4	16%
CCB	1	4%	3	12%	1	4%

*Abbreviations*: *Hyperchol* Hypercholesterolemia, *CAD* coronary artery disease, *PAD* peripheral arterial disease, *BB* beta blockers, *ACEi* ACE inhibitors, *ARB* angiotensin antagonists, *CCB* calcium channel blockers.

**Table 3:** Metabolic features before and after 12 weeks of therapy

	**Metformin**			**Exercise Training**			**Metformin and Exercise Training**		
	**PRE**	**POST**	**P value**	**PRE**	**POST**	**P value**	**PRE**	**POST**	**P value**
Homa	5.1 ± 3.0	3.1 ± 1.3	<0.05	5.5 ± 3.3	3.2 ± 1.1	<0.05	5.6 ± 2.1	3.3 ± 1.2	<0.05
BMI (Kg/m^2^)	29.7 ± 4.8	28.4 ± 4.6	<0.05	28.3 ± 4.7	28.9 ± 5.6	ns	31.8 ± 3.8	29.8 ± 3.8	<0.01
VO2 (l/min)	1.5 ± 0.4	1.5 ± 0.3	<0.01	1.4 ± 0.1	1.70 ± 0.5	<0.05	1.7 ± 0.5	1.8 ± 0.4	<0.05
%	70.7 ± 9.9	66.7 ± 8.6	<0.01	67.5 ± 6	82.0 ± 17.9	<0.05	76.3 ± 18.1	84.4 ± 16.8	<0.05
WORK (Watt)	108 ± 32	104 ± 30	Ns	104 ± 18	114 ± 8	<0.05	115 ± 27	137 ± 33	<0.01
AT (l/min)	1.0 ± 0.3	1.0 ± 0.2	Ns	0.9 ± 0.1	1.1 ± 0.1	<0.05	1.09 ± 0.3	1.05 ± 0.15	<0.05
VE/VCO2	28.2 ± 2.4	27.8 ± 3.3	Ns	22.4 ± 3.2	23.7 ± 1.9	Ns	23.2 ± 2.3	22.2 ± 1.6	<0.05
VO2/WORK	9.5 ± 1.3	9.2 ± 0.8	Ns	9.7 ± 0.9	9.43 ± 3.3	Ns	9.3 ± 1.3	9.3 ± 0.8	Ns

*Abbreviations*: *HOMA* Homeostasis Model Assessment, *BMI* Body Mass Index, *VO2peak* Maximum oxygen uptake, *% VO2* Maximum oxygen uptake in percentage compared to normal values, *Work* Maximum work, *AT* Anaerobic threshold, *VE max* Ventilation at maximum exercise, *VCO2* carbon dioxide production, *VO2/Work* Oxygen uptake-Work rate relationship, *VE/VCO2* Ventilatory equivalent for carbon dioxide.

Of the initial 75 patients, 5 from the Ex group and 4 from the MEx group were excluded from the study as they were unable to regularly follow the physical training program (training attendance <70%).

Variations of the main parameters examined after 12 weeks of therapy in the 3 groups are reported in Table 3. After 12 weeks of treatment body mass index (BMI) was significantly lower in the MEx group (CI 95% 29.8 [27.2 to 30.4] vs 31.8 [30.2 to 33.4]; p < 0.05) and in the M group (CI 95% 28.4 [26.6 to 30.2] vs 29.7 [27.8 to 31.6]; p < 0.05) whereas an insignificant increase in BMI was observed in the Ex group (CI 95% 28.9 [26.5 to 31.3] vs 28.3 [26.2 to 30.4]; p = ns). Furthermore, a significant reduction in HOMA-IR was observed in all 3 groups (Table 3) with no significant difference in the amount of reduction between groups (Table 4).

**Table 4:** Differences between metabolic and anthropometric features after and before 12 weeks of therapy

				**P value**		
	**M**	**MEx**	**EX**	**M vs MEx**	**M vs EX**	**MEx vs EX**
Homa	-1.98 [-2.87 to -1.08]	-2.21 [-3.34 to -1.08]	2.31 [1.60 to 3.02]	ns	ns	ns
BMI (Kg/m^2^)	-1.35 [-2.27 to -0.43]	-1.99 [-2.24 to -1.74]	0.67 [-0.01 to 1.35]	ns	< 0.01	< 0.01
VO2 (l/min)	-0.09 [-0.15 to -0.03]	0.19 [0.05 to 0.33]	0.26 [0.05 to 0.47]	< 0.01	< 0,01	ns
%	-4.70 [-7.31 to -2.1]	8.11 [1.37 to 14.85]	14.42 [9.31 to 19.53]	< 0.01	< 0.01	ns
WORK(Watt)	-3.72 [-9.85 to -2.41]	21.85 [12.52 to 31.17]	10.22 [1.45 to 18.99]	< 0.01	< 0.01	ns
AT (l/min)	-0.06 [-0.14 to 0.02]	-0.04 [-0.13 to 0.05]	0.18 [0.15 to 0.22]	ns	< 0.01	< 0.01

*Abbreviations*: *HOMA* Homeostasis Model Assessment, *BMI* Body Mass Index, *VO2* Maximum oxygen uptake, *% VO2peak* Maximum oxygen uptake in percentage compared to normal values, *Work* Maximum work, *AT* Anaerobic threshold.

The variations of principal metabolic, anthropometric and CPET values before and after the 3 treatments and the differences between groups are reported in Table 4. Ex group showed improvement in the primary endpoint (peak oxygen consumption) compared with M group, whereas MEx group does not improve O2 consumption with respect to Ex group. The same behavior has been observed for the Work. Moreover only Ex group showed an improvement in the Aerobic Threshold with respect to M and MEx groups (Table 4).

Evaluation of traditional echocardiographic parameters did not highlight statistically significant differences between the groups before or after the 3 therapies (data not shown).

CPET highlighted a slight but significant reduction of peak VO2 (1.45 ± 0.34 l/min vs 1.54 ± 0.40 l/min; p < 0.01) in the M group while a significant improvement was seen in the MEx group (1.84 ± 0.38 1/min vs 1.65 ± 0.45 vs p < 0.01) and in the Ex group (1.70 ± 0.51 vs 1.44 ± 0.12 p < 0.05). These trends were also seen when peak VO2 values were considered with respect to predicted values.

At the same time, an insignificant reduction in work was highlighted in the M group, in contrast with a significant increase of the same parameter in the MEx group and in the Ex group (Table 3). The anaerobic threshold was significantly increased in both the MEx group and the Ex group (Table 3).

**Figure 2:**
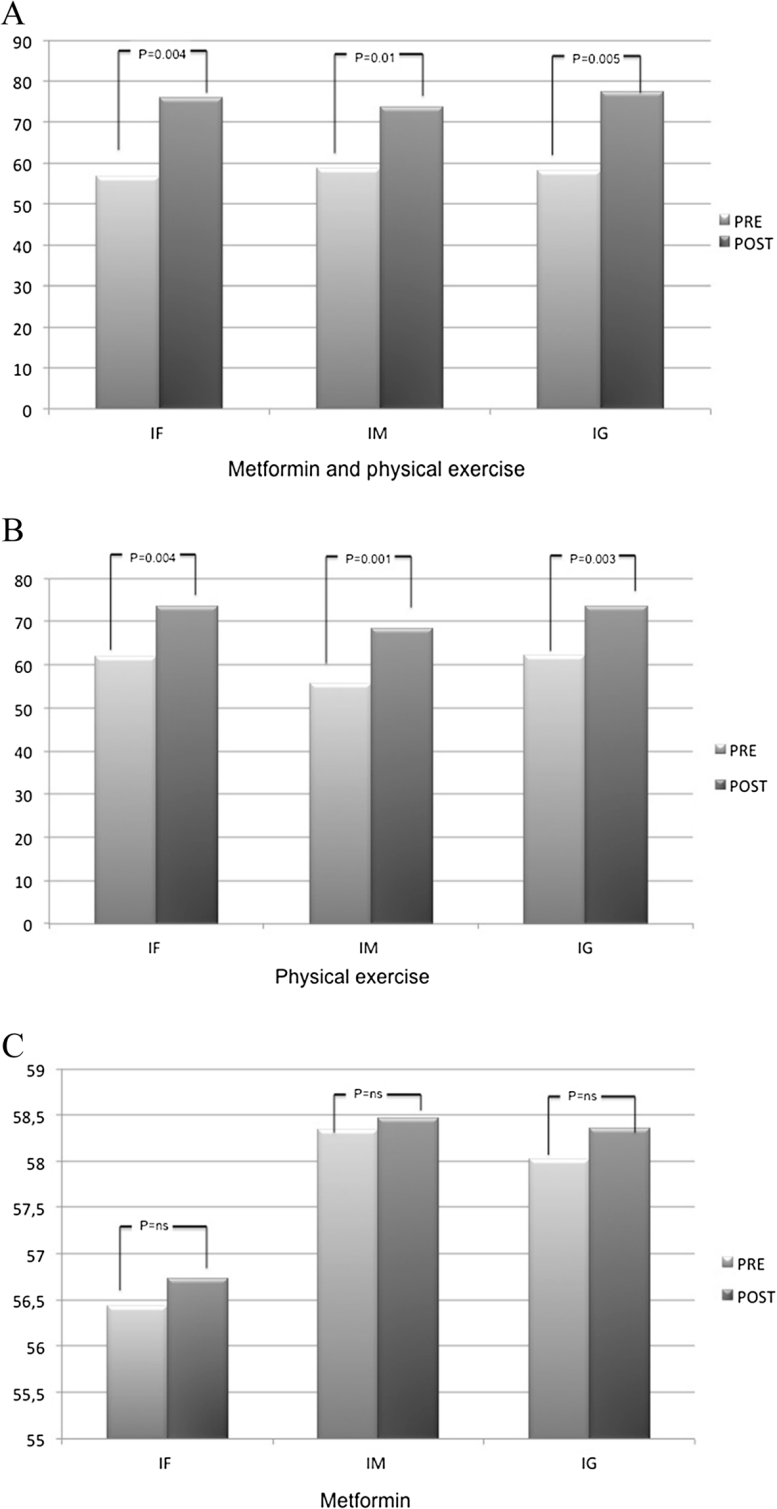
Effects of exercise associated with metformin (panel A), of supervised exercise training alone (panel B) and of Metformin therapy alone (panel C) on physical health index (IF), mental health index (IM) and general health index (GI) after 12 weeks of treatment.

With respect to the secondary endpoint, after 12 weeks of therapy MEx group showed a significant improvement in physical (56.84 ± 19 vs 76.11 ± 14; p 0.004) mental (58.75 ± 18 vs 73.68 ± 17; p 0.01) and general (58.33 ± 19 vs 77.29 ± 16; p 0.005) (Figure 2, panel A) health indexes was observed. Analogously, a significant improvement in physical (61.9 ± 13 vs 73.56 ± 10; p 0.004) mental (55.8 ± 20 vs 68.33 ± 18; p 0.001) and general (62.14 ± 17 vs 73.70 ± 12; p 0.003) health indexes was also witnessed in Ex group (Figure 2, panel B). However, no variations in physical (56.44 ± 19 vs 56.73 ± 18; p NS) mental (58.35 ± 19 vs 58.46 ± 18; p NS) and general (58.03 ± 19 vs 58.36 ± 18; p NS) health indexes were observed in M group (Figure 2, panel C).

No significant differences emerged between variations in physical, mental and general well-being indexes observed in the MEx group (PI: 19.26 ± 14 vs 11.66 ± 11; p = NS – MI: 14.93 ± 14 vs 12.53 ± 10; p = NS – GI: 18.95 ± 15 vs 10.74 ± 12; p = NS), when compared to those seen in Ex group.

## Discussion

Our study found that the IR patients show a reduced CP performance compared with the normal population. Most importantly with respect to the primary endpoint physical exercise treatment alone produce an improvement in oxygen consumption compared with MET therapy alone, whereas MET in association with physical activity does not improve O2 consumption in comparison to therapy with physical exercise alone. Moreover only physical exercise treatment alone produce an improvement in the Aerobic Threshold with respect to MET therapy alone and MET in association with physical activity.

The data referring to the secondary endpoint evidenced that physical exercise, alone or in combination with MET, is superior to MET treatment alone in improving all indices of HRQoL, whether physical or mental. We found no differences on the indices of HRQoL between physical exercise alone and in combination with MET.

In addition, our study confirmed that the treatment with MET decreases the peak VO2 and the ability to work, as already demonstrated by our group in a previous study [9]. Exercise not only improved the CP performance when used alone, but it was able to cancel the negative effect of MET, when used in combination with the drug.

Peak VO2, the maximum capacity of the body to use oxygen, identifies the highest potential for an individual to perform aerobic work. This parameter is influenced by age, sex and level of training, as well as by the presence of disease or drugs capable of affecting any one of its components.The use of exercise capacity as a powerful prognostic factor in normal subjects is widely acknowledged. After adjustment for age, peak exercise capacity measured in metabolic equivalents was the strongest predictor of the risk of death in healthy populations [22]: each metabolic equivalents increase in exercise capacity conferred a 12 percent improvement in survival. A reduced peak VO2 at baseline in the subjects investigated in the present study may be interpreted as a depressive effect induced by IR *per se* on the CP function, as evidenced in our previous experience [9]. In agreement with this hypothesis, a recent community-based study showed that IR predicts the incidence of heart failure, independently of the known risk factors, including diabetes [23].

Several animal models have shown a myocardial metabolic incompetence which could lead to an IR cardiomyopathy [23]. In fact, IR determines in myocytes an energy inefficiency as a result of an increased use of fatty acids, energetically less efficient when compared to glucose. Furthermore, myocytes are unable to utilize glucose in situations of stress, as occurs in healthy subjects. These mechanisms, together with the endothelial dysfunction seen in IR subjects [24], can contribute to myocardial dysfunction [25,26] and the reduction of CP performance that we observed.

In the Diabetes Prevention Program, MET showed the ability to slow the progression from a state of impaired glucose tolerance to T2DM [27,28]. However, several adverse events were attributed to the drug. MET was recognized responsible for a reduction in oxygen consumption in healthy individuals without IR in comparison with controls treated with placebo [8]. In vitro studies found that MET exerts its anti-diabetic effects through inhibition of complex 1 of the mitochondrial respiratory chain (electron transfer from NADH to coenzyme Q 10) [29,30]. This inhibition can slow down the transfer of reducing equivalents during the Krebs cycle and limit the ability of oxidative metabolism. In large muscle groups, the mitochondrial reserve may be used to some extent during exercise. However, if an inhibition of complex 1 reduces this reserve, a critical decrease in CP performance can result. Our previous data showed that MET reduces the average consumption of oxygen for a small but significant extent in IR subjects [9]. It is of particular interest to note that this depressive effect does not manifest itself in all subjects, being present only in individuals with lower degree of IR, while the CP performance is improved in patients with high and very high IR.

Results from the DPP [5] and other similar studies demonstrated that changes in lifestyle, such as a proper diet and increased physical activity, play a crucial role in preventing or slowing the progression from a state of IR to a frank diabetes [31,32]. In the presence of this evidence, a combination of lifestyle changes and MET therapy has been suggested as the best strategy to control IR and prevent diabetes. The data currently available suggest a benefit of the association in terms of weight loss [33], but a little if any effect in reducing IR [34], These findings, although preliminary, demonstrate how the clinical effects of a combination of exercise and MET are complex and difficult to determine in advance. They confirm the general assumption that exercise-drug interactions cannot be predicted from their individual effects and should be considered systematically to provide information usable in the field of public health. Malin et al. indeed demonstrated that exercise and MET are both able to improve insulin sensitivity after 12 weeks of therapy, in men and women with pre-diabetes. But the addition of MET to physical training did not improve IR and, rather, could have obscured the full benefit of exercise [12].

In the present study, both the exercise associated with MET and MET alone led to a significant weight loss, while the exercise used alone did not have this effect. These data agree with those previously published in the literature, according to which MET is able to determine by itself a weight loss. Our findings demonstrate, to our knowledge for the first time, that treatment with MET in combination with physical exercise is not superior to exercise alone, in terms of improving CP performance. Conversely, the addition of a tailored and supervised training program in subjects with IR is crucial to reverse the adverse effects of MET on the oxygen consumption and allow a greater adherence to therapy.

Various and mostly little-known mechanisms underlie the MET/exercise interaction and make the effects of this therapeutic association unpredictable. Both exercise and MET act on protein kinase activated by the AMP (AMPK) expression [35] and on the transduction pathway of the AMPK–eNOS signal. Metformin, an AMPK activator which can act as an exercise mimetic [36], had been shown to improve exercise in women with angina [37] and to improve the endothelial flow reserve [38].

In our study, as previously observed in the study of Malin et all [12], BMI was significantly lower in the MEx group and in the M group and not in the Ex group. This result may be related to a different effect of metformin and exercise on fat mass and lean body mass distribution.

The last, but not less important remark concerns the net effect of these therapies on HRQoL related to physical, mental and general well-being. The data obtained allow us to confirm the benefit of exercise, without further advantages obtained by its combination with MET. It is our belief that this latest finding is extremely important in a population of relatively young subjects, which, although at high risk of developing diabetes and cardiovascular disease, must however be considered healthy. When MET is employed, it should always be associated with physical exercise to counteract the negative effects of drug therapy on CP performance, contribute to improving the HRQoL and increase patient compliance, which, as is known, is a crucial component in their clinical management.

### Limitations

The main limitations of the study are related to the study design. A single blinded design was chosen due to the difficulties of concealing the exercise. Moreover since the primary endpoint of our study was to verify the effect of exercise alone or in combination to metformin on the maximal aerobic capacity in comparison with the patients not performing exercise a “per protocol analysis” was chosen as more appropriate. Although removing the patients who were unable to regularly follow the physical training program from the final analysis introduces potential bias.

## Conclusions

The objective of this study was to evaluate the combined effect of MET and supervised physical exercise on CP performance and the HRQoL in subjects with IR. The negative CP effects induced by MET alone may be compensated by the association with supervised physical activity. Exercise training alone, when personalized and supervised, produces effects similar to the combination of MET and exercise in terms of CP performance and HRQoL. Given the lacking evidence of a benefit from previous studies of the association of MET and exercise in the control of IR, we confirm that a programmed and tailored training should be the first choice treatment IR patients. Further studies are warranted to demonstrate the real long-term clinical benefit of MET and supervised physical exercise alone or associated.

## Abbreviations

MET: Metformin; M: Metformin therapy group; MEx: Metformin with exercise training group; Ex: Exercise training group; HRQoL: Health-Related Quality of Life; IR: Insulin resistance; CP: Cardiopulmonary; IGT: Impaired glucose tolerance; IFG: Impaired fasting glucose; HOMA: Homeostatic model assessment; BMI: Body mass index; LVEF: Left ventricle ejection fraction; CPET: Cardiopulmonary exercise test; VO2peak: Maximum oxygen uptake; AT: Anaerobic threshold; VE max: Ventilation at maximum exercise; VCO2: carbon dioxide production; VO2/Work: Oxygen uptake-Work rate relationship; VE/VCO2: Ventilatory equivalent for carbon dioxideproduction; PCS: Physical component summary; MCS: Mental component summary.

## Competing interests

The authors declare that they have no competing interests.

## Authors’ contribution

CC Study design, manuscript writing. SN Study design, manuscript writing, Data collection. LC Data collection and Data analysis. AB Echocardiography, Data analysis. MD Echocardiography, Data analysis. FO Data collection, clinical examination. SB Data collection, clinical examination. EC Patients recruitment, study design. MGB Study design, Data interpretation. GM Study Design, Manuscript writing, Data interpretation. All authors read and approved the final manuscript.
